# Could Ovarian Veins Be the Origin of Most Retroperitoneal Leiomyosarcomas?

**DOI:** 10.1002/iju5.70067

**Published:** 2025-06-26

**Authors:** Cuneyt Kayaalp

**Affiliations:** ^1^ Istanbul Atlas University Istanbul Turkiye

**Keywords:** leiomyosarcoma, retroperitoneal sarcoma, vena cava

Dear Editor,

I have read with interest the case report of Iwagami et al. “Internal High Echoes Can Suggest the Possible Resection of Ovarian Vein Leiomyosarcoma: A Case Report” [1], which describes a rare patient with leiomyosarcoma originating from the right gonadal vein. The authors have clearly defined the radiological continuity of the tumor with the gonadal vein and revealed its relationship with adjacent structures such as the vena cava and duodenum.

The authors have successfully revealed that the gonadal vein is the source of a retroperitoneal leiomyosarcoma, I congratulate them. The origin of the majority of retroperitoneal leiomyosarcomas cannot be determined. But there are some indirect findings that suggest that a significant part of it may actually be originated from gonadal veins.

The findings supporting this under‐diagnosis hypothesis are:

1. Anatomy: The gonadal veins pass through the retroperitoneum and flow directly into the vena cava or renal vein. The majority of vena cava leiomyosarcomas are observed in the suprarenal part where the renal veins drain. Vascular leiomyosarcoma is most often seen in the suprarenal vena cava and secondly in the renal veins. More leiomyosarcomas are observed in the vessels where sex hormones are transported [2].
2. Delayed diagnosis: Vascular leiomyosarcomas are generally asymptomatic in the early stages and are usually diagnosed when the tumors reach significant sizes and it becomes difficult to determine their origin [3, 4]. In the case of Iwagami et al., the diagnosis of retroperitoneal leiomyosarcoma at a relatively lower size (< 6 cm) and helped with its origin.
3. Female gender: Retroperitoneal leiomyosarcomas appear more often in women. Leiomyosarcoma is more common in estrogen‐carrying vessels [2]. In the body, leiomyosarcoma most often develops in the uterus.

Awareness is needed for future studies to evaluate gonadal veins as a potential source of retroperitoneal leiomyosarcomas of uncertain origin. For this reason, I believe that retroperitoneal leiomyosarcomas, whose origin has been definitively established, although rare, are important, as in this case.

The fact that the gonadal vein has a possible origin in retroperitoneal leiomyosarcomas could potentially change treatment strategies. Immunohistochemical profile examinations in pathological examinations can be a guide in this regard.

## Conflicts of Interest

The author declares no conflicts of interest.

## Linked Articles

This article is linked to Iwagami et al. paper. To view this article, visit https://doi.org/10.1002/iju5.70025.
